# Oral LPS Dosing Induces Local Immunological Changes in the Pancreatic Lymph Nodes in Mice

**DOI:** 10.1155/2019/1649279

**Published:** 2019-03-06

**Authors:** Pernille Kihl, Lukasz Krych, Ling Deng, Anna Overgaard Kildemoes, Ann Laigaard, Lars Hestbjerg Hansen, Camilla Hartmann Friis Hansen, Karsten Buschard, Dennis Sandris Nielsen, Axel Kornerup Hansen

**Affiliations:** ^1^Department of Veterinary and Animal Sciences, University of Copenhagen, Grønnegårdsvej 15, 1870 Frederiksberg C, Denmark; ^2^Department of Food Science, University of Copenhagen, Rolighedsvej 26, 1958 Frederiksberg C, Denmark; ^3^Department of Environmental Sciences, University of Århus, Frederiksborgvej 399, 4000 Roskilde, Denmark; ^4^Bartholin Institute, Rigshospitalet, Ole Måløesvej 5, 2200 Copenhagen N, Denmark

## Abstract

Lacking the initial contact between the immune system and microbial-associated molecular patterns (MAMPs), such as lipopolysaccharides (LPS), early in life, may be regarded as one of the causal factors of the increasing global increase in the incidence of autoimmune diseases, such as type 1 diabetes (T1D). Previously, a reduced incidence of T1D accompanied by dramatically increased abundances of both the mucin-metabolising bacterium *Akkermansia muciniphila*, and LPS-carrying Proteobacteria was observed, when vancomycin was given to pups of nonobese diabetic (NOD) mice. While the T1D incidence reducing effect of *A*. *muciniphila* has been shown in further studies, little is known as to whether the increased abundance of LPS-carrying bacteria also has a protective effect. Therefore, we fed NOD pups with *Eschericia coli* LPS orally from birth to weaning, which decreased the gene expressions of *TNFα*, *IL-10*, *IL-6*, *IFNγ*, *IL-1β*, *IL-2*, *IL-4*, and *FoxP3* in the pancreatic lymph nodes, while the same gene expression profile in the spleen was unaffected. However, no significant difference in the incidence of T1D, gut microbiota composition, or ileum expression of the genetic markers of gut permeability, *Claudin8*, *Occludin*, *Zonulin*-*1 (Tjp1)*, *Claudin15*, *Muc1*, and *Muc2* were observed in relation to LPS ingestion. It is, therefore, concluded that early life oral *E*. *coli* LPS has an impact on the local immune response, which, however, did not influence T1D incidence in NOD mice later in life.

## 1. Introduction

The hygiene hypothesis proposes early life contact between microbes and the immune system to be essential for a proper later life balance between adaptive tolerance and reaction to inflammatory challenges [1]. With some modifications to the original hypothesis [2], several studies have strengthened the idea that the first encounter between mucosal surface proteins and various microorganisms drives both B and T and particularly regulatory T cell (T_reg_) expansion [3, 4]. Failure of this is a likely explanation of the increasing global increase in the incidence of type 1 diabetes (T1D), multiple sclerosis, and other autoimmune diseases, as well as asthmas and allergies [5–9]. In T1D, molecular dysfunctions lead to the pancreatic infiltration with islet antigen-specific cells (insulitis), which is initiated by neutrophils, dendritic cells, and macrophages, and continued with T and B lymphocyte reactivity, which autonomously attack the insulin-producing pancreatic beta cells [10]. In humans [8] as well as in the most common T1D animal model, the nonobese diabetic (NOD) mouse [10, 11], early-life gut microbiota dysbiosis seems to have an important influence on the onset and progression of T1D [12, 13]. In germ-free NOD mice an accentuated degree of insulitis, however without a higher incidence of T1D, has been observed [14], while there are also reports of the opposite, i.e., unchanged insulitis but a higher incidence of T1D in germ-free NOD mice [15]. Therefore, it has been hypothesized by some that only a very specific microbiota composition is diabetogenic [16]. Cell walls of various bacteria carry innately immune-stimulating microbial-associated molecular patterns (MAMPs), such as lipopolysaccharides (LPS) [17, 18]. When recognized by Toll-like receptor 4 (TLR4) [19], LPS initially induces production of inflammatory cytokines, e.g., TNF*α* and IL-1 [20], and later on interferon *β* [21] through nuclear factor *κ*B activation [22]. Recently, we were able to reduce the T1D incidence by orally feeding the antibiotic vancomycin to NOD pups from birth to weaning, which dramatically propagated Verrucomicrobia and Proteobacteria, and reduced all other bacterial phyla [23]. The only Verrucomicrobia genus in mice, *Akkermansia muciniphila*, influences mucosal immune response and degrades the mucin layer by feeding on mucin [24], while Proteobacteria contain a high concentration of LPS in its cell walls [8]. The T1D protective effect of vancomycin [23] could therefore mechanistically have its background in a combination of the mucin degrading effect of *A. muciniphila* and increased TLR4 stimulation by LPS. Transfer of *A*. *muciniphila* from a low to a high incidence NOD mouse colony delays T1D development [25], and in streptozotocin diabetic rats, *A*. *muciniphila* supplementation suppresses inflammation [26]. However, monocolonization of *A*. *muciniphila* in germ-free NOD mice has no effect on type 1 diabetes incidence [27] suggesting that the simultaneous presence of other bacteria, as e.g., the LPS rich Proteobacteria, are necessary for *A. muciniphila* to exert a protective effect. It has previously been observed that human T1D patients have increased levels of serum LPS [28], and that injection of LPS in the prediabetic phase of NOD mice may delay the onset of T1D [29]. Early life exposure to LPS and especially the balance between LPS of *Bacteroides* and Enterobacteriaceae (Proteobacteria) origin is linked to the occurrence of T1D-associated antibodies in genetically susceptible human toddlers [8]. Consequently, it is relevant to study, which impact early-life ingestion of LPS may have on the pancreatic immune response and T1D development, and whether LPS can be regarded as a part of a mechanistic explanation of the protective effect of vancomycin in NOD mice. As it has been shown that the LPS content of laboratory rodent diets influence the development of regulatory T cells [4] and consequently has a modest impact on rodent models of type 2 diabetes [30], it may also be relevant to elucidate whether it influences the NOD mouse as a model for T1D. We, therefore, hypothesized that administration of LPS in early life would reduce proinflammatory gene expression in the pancreatic lymph node (PLN) and subsequently T1D incidence in NOD mice.

## 2. Material and Methods

### 2.1. Mouse Experiments

Experiments were approved by the Animal Experiments Inspectorate, Ministry of Environment and Food, Denmark (License 2012-15-2934-00256–C1) in accordance with the EU Directive 2010/63/EU on the Protection of Vertebrate Animals used for Experimental and Other Scientific Purposes and the Danish Animal Experimentation Act (LBK 474 from 15/05/2014). 40 (18 female and 22 male) C57BL/6NTac (B6) and 34 female NOD/MrkTac mice were bred in-house on breeding pairs purchased from Taconic (Germantown, USA) and housed in our AAALAC-accredited barrier-protected and microbiologically monitored [31] facility with a 12-hour light-time schedule, in open cages with *ad libitum* food and water access. Purified *E. coli* 0127: B8 LPS (Sigma-Aldrich, Brøndbyvester, Denmark) was diluted in phosphate-buffered saline (PBS) (0.2 mg/mL) and administered orally with a pipette every other day from birth till four weeks of age with 10 *μ*l and 20 *μ*l pr. mouse (B6: *n* = 18 and NOD: *n* = 11) for the first two and the last two weeks, respectively. An equal dose of PBS was administrated to the control mice (B6: *n* = 22 and NOD: *n* = 23). All mice were monitored for overall well-being throughout the study and weighed weekly. At four weeks of age, the B6 mice were weighed and anaesthetized with Hypnorm (fentanyl and fluanisone, VetaPharma, Leeds, England) and Dormicum (midazolam, F. Hoffmann-La Roche, Basel, Switzerland) dosed subcutaneously with 0.5 mL/100 g of body weight as a 1 : 1 : 2 aqueous solution. The total blood volume was collected from the retro-orbital plexus into sterile Eppendorf tubes (Eppendorf, Germany), and blood was stored on ice for approximately one hour before being centrifuged (500 x g, 10 min). Hereafter, the mice were sacrificed by cervical dislocation prior to harvesting feces, the pancreatic lymph nodes (PLN), the spleen, and the ileum. Samples were stored at -80°C until analysed. Tail-vein blood glucose was monitored in NOD mice weekly until 30 weeks of age, and a blood glucose value of ≥12 mmol on two consecutive measurements was diagnosed as T1D. If their overall condition was deteriorating, they were euthanized.

### 2.2. Analyses

LPS concentration in serum was analysed using a Limulus Amebocyte Lysate (LAL) chromogenic endpoint assay for endotoxin detection (HIT302 edition 10-16, Hycult Biotech, Netherlands). The samples were diluted 1 : 10, run in duplicates, with an extra control sample with no LAL reagent added. Samples were analysed according to the manufacturer's instructions, with the addition of a heating step (70°C for 5 min) prior to adding the samples to the wells, to neutralize endotoxin inhibiting compounds.

Total fecal bacterial DNA was extracted using the PowerSoil® DNA Isolation Kit (MO BIO Laboratories, Carlsbad, CA), following the instructions of the manufacturer with minor modifications. Samples were placed into the PowerBead tubes and heat-treated at 65°C for 10 min and then at 95°C for 10 min prior DNA extraction. Subsequently, solution C1 was added and bead-beating performed in FastPrep (MP Biomedicals, Santa Ana, CA, USA) using 3 cycles of 15 s each, at a speed of 6.5 ms. The gut microbiota composition was determined using tag-encoded 16S rRNA gene (V3-V4 regions) MiSeq-based (Illumina, San Diego, CA) high-throughput sequencing. DNA extraction, storage conditions, and sequencing library preparation were all conducted as previously described [32].

Gene expressions of the spleen, pancreatic lymph nodes (PLN), and ileum were analysed by quantitative PCR (qPCR). Samples were homogenized with FastPrep in 500 *μ*l lysis buffer MagMAX™-96 Total RNA Isolation Kit (AM1830, Thermo Fisher Scientific, Herlev, DK) with 0.7% *β*-mercapthoethanol and glass beads and then stored at -20°C prior to RNA purification (AM1830, and MagMAX™ Express magnetic particle processor, Thermo Fisher). cDNA synthesis, qPCR, and data management were performed as described previously [33], i.e., the expression of target genes were normalized to the reference gene *Actinβ*, (Δ*C*_*T*_ = *C*_*T*(*target*)_–*C*_*T*(*reference*)_). Fold change in gene expression was calculated as 2^-*Δ*ΔCT^, where ΔΔ*C*_*T*_ = Δ*C*_*T*(*sample*)_ − Δ*C*_*T*(*calibrator*)_ and where the mean Δ*C*_*T*_ of samples from the control mice was used as a calibrator. Genes analysed in the spleen and PLN were *IL-18*, *TNFα*, *IL-10*, *IL-6*, *IFNγ*, *IL-1β*, *IL-12β*, *IL-5*, *IL-2*, *IL-4*, *IL-17α*, and *Foxp3*. Genes analysed in the ileum were *Claudin8*, *Occludin*, *Zonulin*-*1 (Tjp1)*, *Claudin15*, *Muc1*, and *Muc2*.

### 2.3. Statistical Analyses

Comparisons of relative gene expression levels were done with one-tailed Student's *t*-test, if d'Agostino-Pearson test showed a Gaussian distribution, and *F*-test showed equal variances, and if not with one-tailed Mann-Whitney test, and analysis of interference by sex (B6 mice), was done by applying a two-way ANOVA. Cumulative diabetes incidence was calculated using the Kaplan–Meier estimation, while statistical significance was evaluated by the logrank test. All statistical tests were done with GraphPad Prism 7.0 (GraphPad Software, La Jolla, USA). For gut microbiota characterisation, Illumina MiSeq-platform generated 16S rRNA gene amplicons (V3-V4regions) were analysed as previously described [34].

## 3. Results

### 3.1. LPS Influences Inflammatory State in the PLN in B6 Mice, While It Does Not Influence the T1D Incidence in NOD Mice

**Figure 1:**
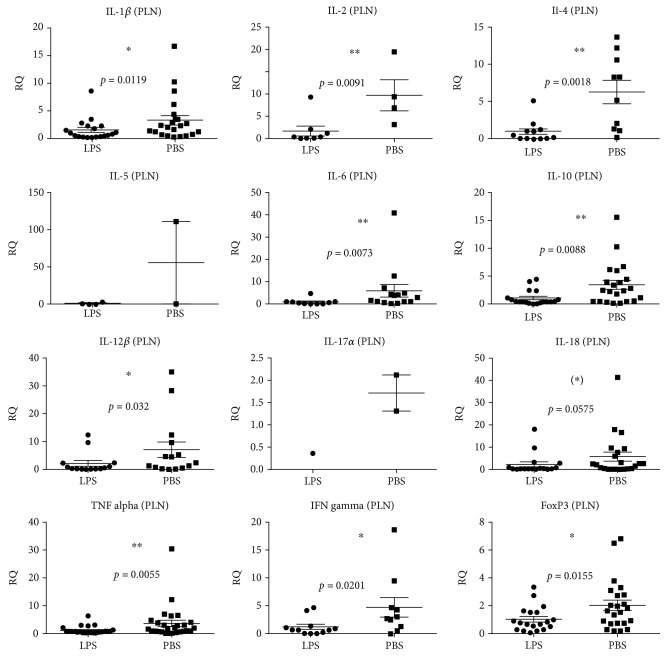
Cytokine and regulatory T cell (*FoxP3*) gene expressions in the pancreatic lymph nodes (PLN) of 40 (18 female and 22 male) four-week-old C57BL/6NTac mice given either LPS (*n* = 18) or PBS (*n* = 22) orally daily from birth until weaning at four weeks of age. Mean and SD are shown. ^∗^*p* < 0.05; ^∗∗^*p* < 0.01; ^∗^tendency *p* = 0.05–0.1.

**Figure 2:**
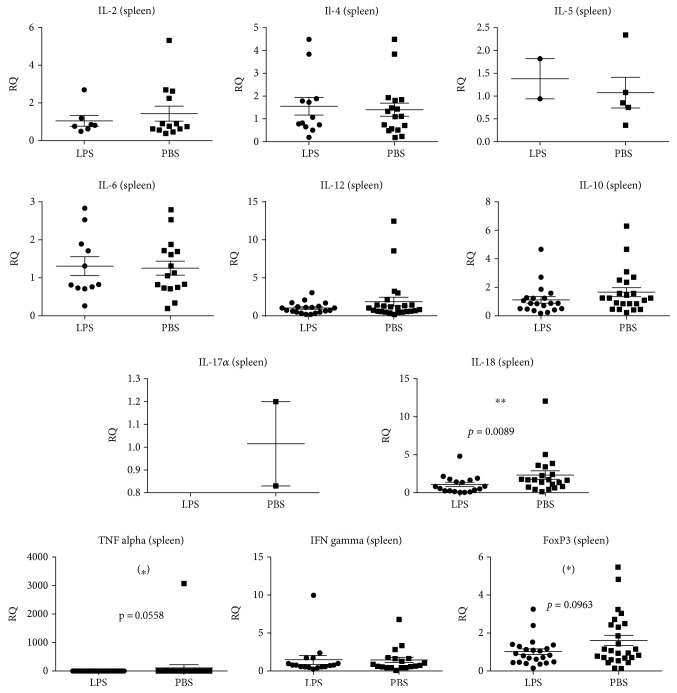
Cytokine and regulatory T cell (*FoxP3*) gene expressions in the spleen of 40 (18 female and 22 male) four-week-old C57BL/6NTac mice given either LPS (*n* = 18) or PBS (*n* = 22) orally daily from birth until weaning at four weeks of age. Mean and SD are shown. ^∗∗^*p* < 0.01; ^∗^tendency *p* = 0.05–0.1.

The immune stimulatory effect of LPS from birth to four weeks of age was investigated by measuring the relative gene expression of cytokines in B6 mice at four weeks of age. In the PLN, LPS administration significantly decreased the expression of the majority of pro- and anti-inflammatory cytokine genes analysed, i.e., *IL-1β*, *IL-2*, *IL-4*, *IL-6*, *IL-10*, *IL-12β*, *TNFα*, *IFNγ*, and *FoxP3* (Figure 1). For *IL-1β*, *IL-2*, *IL-4*, *IL-6*, *IL-10*, *IL-12β, TNFα*, *IFNγ*, and *FoxP3*, the control groups had a significantly larger interindividual variation (Figure 1). Sex had no significant impact. LPS did not seem to have a strong influence on the systemic level of inflammation, as only *IL-18* was significantly downregulated in the spleen, while there was a tendency towards downregulation of *FoxP3* and *TNFα* . However, as it was the case in PLN, *IL-12β*, *IL-18*, *TNFα*, and *FoxP3* (*p* = 0.0019) also expressed a higher variation in the spleens of the control groups (Figure 2).

**Figure 3:**
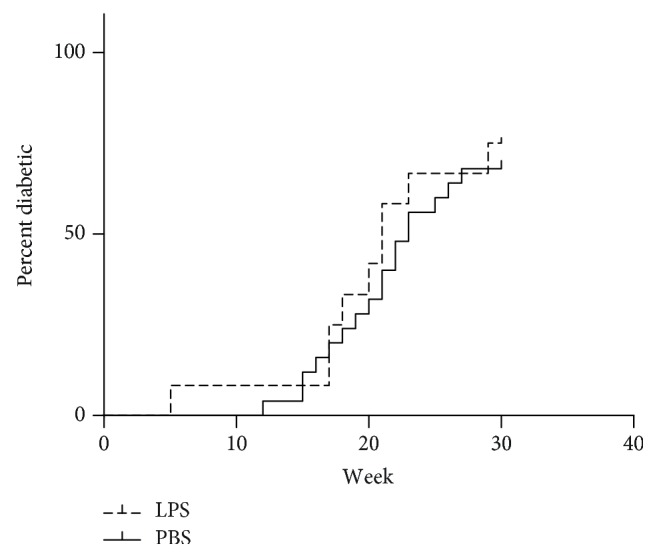
Incidence of type 1 diabetes diagnosed as three consecutive blood values of glucose >12 mmol/l in NOD/MrkTac mice given either LPS (*n* = 18) or PBS (*n* = 22) orally daily from birth until weaning at four weeks of age and observed until 30 weeks of age.

75% of NOD mice, which had received oral LPS for the first four weeks of life developed T1D compared to 68% of the PBS-treated control mice (Figure 3), which was not significantly different. NOD mothers of the pups on LPS treatment appeared to become diabetic faster and then being less able to take care of their pups, resulting in small pups, which in some cases had to be euthanized for humane reasons. Three moms in the LPS group had to be euthanized or died prior to weaning of the pups, while there were no such incidences in the PBS group.

### 3.2. In the Gut of B6 Mice, *Prevotella* Spp. Were Downregulated after LPS Administration, While Permeability Was Not Influenced

**Figure 4:**
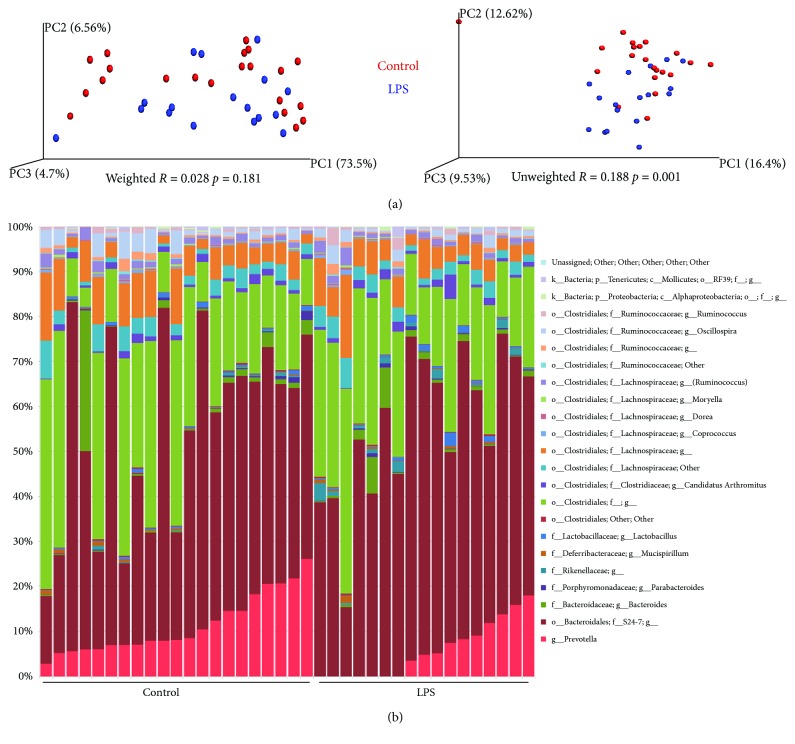
Weighted and unweighted principal component analysis plots showing microbiota clustering (a) and bar chart demonstrating relative abundances of genera (b) after 16S sequencing of feces of 40 (18 female and 22 male) four-week-old C57BL/6NTac mice given either LPS (*n* = 18) or PBS (*n* = 22) orally daily from birth until weaning at four weeks of age.

LPS administration did not induce any significant difference in the overall gut microbiota composition (Figure 4). The abundances of some genera were influenced by the LPS (Table 1), but among these, only *Prevotella* spp. were observed with a high abundance, i.e., 11.2% in the PBS-treated control mice, while only 5.7% in the LPS-treated mice (Table 1). There were no indications of increased amounts of LPS entering the blood stream, as LPS-administered mice did not have more LPS in serum than the control mice (data not shown), which also matched the finding that none of the genetic markers of gut permeability, *Claudin8*, *Occludin*, *Zonulin*-*1 (Tjp1)*, *Claudin15*, *Muc1*, and *Muc2* were expressed differently in the ileum in relation to LPS administration (data not shown).

## 4. Discussion

As hypothesized, administration of LPS in early life reduced both anti- and proinflammatory cytokine gene expressions in PLN. The impact was far less or absent in the spleen, which indicates that the effect of LPS is local and not systemic, supporting previous observations on bacterially derived activation of the immune system [35]. This also matches the fact that there was not a surplus of LPS which entered the blood stream due to the LPS administration, and that gut permeability was unchanged. An increased inflamed milieu in the PLN has been observed prior to onset of insulitis [36], which is considered important for the attraction and activation of the autoreactive T cells that circulate in the host thereby initiating the T1D development [37]. Such local effects of LPS on the cytokine milieu in the PLN, with no systemic indication, require a homing specifically to the PLN, as it has been proposed previously [38]. It is likely that LPS has the potential to halter production of inflammatory cytokines in the PLN through homing receptors [35]. LPS is known to promote the secretion of several inflammatory cytokines (IL-1, TNF*α*, IFN-*α*, IL-12, IL-15, and IL-18) [39, 40], which to a certain extent are the same cytokines regulated by those genes that we observed downregulated in the PLN after LPS administration. Also, the LPS receptor TLR4 has previously been correlated with increased levels of IL-1*β* and TNF*α* in T1D patients [41]. This may seem contradictory, but it is the concept of the hygiene hypothesis that early-life inflammatory stimulation on a long-term basis activates more regulatory than inflammatory immunity, which is also observed in the low-dose dextran sodium sulphate (DSS) model [42, 43]. So, the timing and dose of LPS exposure is important, indicating a “window of opportunity” for eliciting a protective effect of LPS.

We monitored inflammatory parameters after four weeks of dosing. Obviously, we do not know how early before that the effect would have been measurable. However, in a previous study on older diet-induced obese mice, we were unable to register any impact after only two weeks of oral LPS dosing [30]. It is also apparent from the present and previous studies in the low-dose DSS model that such an early-life inflammatory stimulation does not necessarily have major preventive impact on the development of T1D [29, 44], although one study showed a delayed onset if LPS was injected in the prediabetic phase [29]. As an early-life DSS treatment alleviates symptoms of oxazolone-induced colitis in mice [43], which, in contrast to the Th1 dominated T1D, is dominated by Th2 cells, eventually the LPS effect could be based upon a shift in cell type balance. This is, however, not indicated by our study, as those genes for which the expression was downregulated in PLN were related to different types of T cells. It has previously been observed that human T1D patients have increased levels of serum LPS [28], i.e., while early-life LPS may induce regulatory immunity later in life, LPS stimulation later in life may favour development of inflammatory and autoimmune disease, such as T1D. As injection of LPS in the prediabetic phase of NOD mice seemed to delay the onset of T1D in another study [29], it is difficult to say when the window for a regulatory effect of LPS closes.

The LPS of Bacteroidetes has been found to have severely impaired capacity for immune stimulation relative to LPS from Enterobacteriaceae [8]. A key difference between Finnish and Estonian children with a high T1D incidence from Russian children with a low T1D incidence is that much of the gut LPS in the Finnish and Estonian children is Bacteroidetes-derived, while in the Russian children it derives from *E*. *coli* (Proteobacteria) [8]. Notably, our oral administration of LPS had only minor influences on GM composition. The abundance of *Bacteroides* spp. was low in both groups, while S24-7 belonging to Bacteroidales was high in both groups, but none of them differed between the groups. *Prevotella* also belongs to the phylum Bacteroidetes, and it was observed to be significantly decreased in the gut of mice having received oral LPS, indicating that the LPS dose may have had a suppressing effect on this particular genus. It is also striking that the Betaproteobacteria-containing families, such as Enterobacteriaceae, was below the detection level in all mice, i.e., the endogenic level of *E*. *coli* LPS was very low, indicating that the NOD mice more resemble the Finnish/Estonian children than the Russian children.

It is of course of interest that NOD mothers of the pups on LPS treatment appeared to become diabetic faster than mothers of PBS-treated mice. The most reasonable explanation is that they received an LPS dose from their pups. It is difficult to state, how much this has been. In studies of acute inflammation, mice are dosed with up to 10 mg/kg LPS intraperitoneally [45], and for the moms to get this dose from their pups, they should more or less have eaten everything which was dosed to their pups, which would not be possible. However, much lower doses are known to induce low-grade inflammation in mice [46]. The unintended dosing of the mothers in our study has been oral, and in a previous study, we did not observe dramatic inflammatory responses after a low oral dose of LPS [30]. On the other hand, it cannot be excluded that some of the effects we observed in the pups of the present study were transferred from the mother and were caused by her ingestion of LPS.

In summary, our results demonstrate that although LPS dosing in early life has an impact on the cytokine expressions in the PLN, this was, in this study, not sufficient or of a kind that influenced T1D incidence in NOD mice later in life. Therefore, even though vancomycin leads to dramatically increased abundances of both *A. muciniphila* and LPS-carrying Proteobacteria [23], the protective effect in relation to T1D in NOD mice is more likely to be related to *A*. *muciniphila*, while purified *E*. *coli* 0127: B8 LPS, as used in this study, probably does not play a major role. This is in accordance with recent observations that the sole presence of *A*. *muciniphila* in the microbiota of a colony of T1D prone NOD mice can decrease the incidence of T1D [25].

## Figures and Tables

**Table 1:** Abundances of organisational taxonomic units (OTUs) significantly different in the gut microbiota of C57BL/6NTac mice orally fed with lipopolysaccharides (LPS) for four weeks after birth. P: Phylum; c: class; o: order; f: family; g: genus.

	*p*	False discovery rate *p*	Bonferroni *p*	Control mean	LPS mean
p__Firmicutes; c__Erysipelotrichi; o__Erysipelotrichales; f__Erysipelotrichaceae; g__cc_115	0.001	0.139088256	0.139088256	0.001%	0.005%
p__Bacteroidetes; c__Bacteroidia; o__Bacteroidales; f__Rikenellaceae; g__	0.002	0.156901623	0.313803246	0.201%	1.020%
p__Bacteroidetes; c__Bacteroidia; o__Bacteroidales; f__Prevotellaceae; g__*Prevotella*	0.012	0.510753902	1	11.236%	5.717%
p__Actinobacteria; c__Coriobacteriia; o__Coriobacteriales; f__Coriobacteriaceae; g__	0.015	0.510753902	1	0.001%	0.000%
p__Firmicutes; c__Clostridia; o__Clostridiales; f__Clostridiaceae; g__*Clostridium*	0.026	0.676126791	1	0.007%	0.007%
p__Firmicutes; c__Erysipelotrichi; o__Erysipelotrichales; f__Erysipelotrichaceae; g__*Coprobacillus*	0.035	0.769160931	1	0.013%	0.004%
p__Proteobacteria; c__Alphaproteobacteria; o__Rhizobiales; f__Phyllobacteriaceae; g__	0.042	0.777274747	1	0.001%	0.000%
p__TM7; c__TM7-3; o__CW040; f__F16; g__	0.047	0.777274747	1	0.002%	0.007%

## Data Availability

The data used to support the findings of this study are stored on the University of Copenhagen backup servers, and they are available from the corresponding author upon request.
